# Femoral tunnel length does not impact outcomes following ACL reconstruction using a single‐bundle quadriceps tendon autograft: A systematic review

**DOI:** 10.1002/ksa.12395

**Published:** 2024-07-31

**Authors:** Tess Bracken, Alexandre Veilleux, Hassaan Abdel Khalik, Jansen Johnson, Darren de SA

**Affiliations:** ^1^ Michael G. DeGroote School of Medicine McMaster University Hamilton Ontario Canada; ^2^ Division of Orthopaedic Surgery McMaster University Hamilton Ontario Canada

**Keywords:** anterior cruciate ligament, arthroscopy, autograft, femoral tunnel length, knee, quadriceps tendon

## Abstract

**Purpose:**

To determine whether femoral tunnel length (FTL) affects clinical or functional outcomes following primary Anterior cruciate ligament reconstruction (ACLR) with single‐bundle quadriceps tendon autograft, both with and without a patellar bone block.

**Methods:**

An electronic search of MEDLINE, EMBASE, and Cochrane databases was carried out via OVID. Data pertaining to study characteristics, patient demographics, surgical techniques, femoral tunnel length, and subjective/objective clinical outcomes was abstracted. Studies were stratified into two groups based on FTL; a short femoral tunnel (S‐FT) group of ≤25 mm, and a long femoral tunnel (L‐FT) group of >25 mm. There was a high degree of heterogeneity between studies, prohibiting meta‐analysis.

**Results:**

Seven studies comprising 368 total patients with a mean age of 30.3 years (range: 23.4–34 years) were included for analysis. The S‐FT group included 126 patients and the L‐FT group 242 patients. Both groups demonstrated statistically significant postoperative improvements across both subjective and objective clinical and functional outcomes. Average complication rates were 11.9% (range: 0%–29%) in the S‐FT group and 4.5% (range: 1%–14%) in the L‐FT group. Ranges of re‐rupture rates were 0%–2% and 0%–3% for the S‐FT and L‐FT groups, respectively (n.s.).

**Conclusion:**

Both S‐FT and L‐FT groups demonstrated comparable postoperative outcomes following primary ACLR with single bundle quadriceps tendon autograft. There were slightly superior, although non‐significant, outcomes reported with short femoral tunnel length, however, this may have been confounded by the variation in surgical technique used.

**Level of Evidence:**

Level IV.

**Abbreviations**
ACL: anterior cruciate ligament
ACLR: anterior cruciate ligament reconstruction
AP: anteroposterior
BPTB: bone patellar tendon bone
DB: double bundle
FTL: femoral tunnel length
HT: hamstring tendon
IKDC: international knee documentation committee
L‐FT: long femoral tunnel
LOE: level of evidence
MINORS: methodological index for non‐randomised studies
NR: not reported
PRISMA: preferred reporting items for systematic reviews and meta‐analysis
QT: quadriceps tendon
ROM: range of motion
SB: single bundle
SD: standard deviation
S‐FT: short femoral tunnel

## INTRODUCTION

Anterior cruciate ligament (ACL) tears are one of the most common knee injuries in the United States [39]. ACL reconstruction (ACLR) is an effective treatment option for ACL tears, particularly in cases of joint instability, concomitant meniscal injury, and anticipated future participation in multidirectional pivoting sports [13, 23]. Autografts are typically the preferred graft type with the three most common graft options being bone‐patellar‐bone (BPTB), hamstring tendon (HT), and quadriceps tendon (QT), with BPTB and HT historically being favoured. However, recently the quadriceps tendon (QT) autograft has been increasing in popularity [2, 32, 37]. The QT has been associated with decreased harvest‐site pain compared to BPTB [31, 35], tolerates a higher load to failure [10], and demonstrates clinical and functional outcomes similar or superior to other graft options [15, 19, 38]. Aside from graft choice, there are several other surgical variables that may have a bearing on clinical and functional outcomes following ACLR, namely, femoral tunnel position or length [7].

There is a lack of evidence pertaining to the optimal femoral tunnel length (FTL). Several studies assess femoral tunnel position and length as an outcome of reaming technique. For example, the anteromedial portal technique is associated with a more oblique femoral tunnel position and replicates the native anatomic position of the ACL with greater ease [5, 7]. Conversely, multiple studies demonstrated that the transtibial technique creates the longest and most vertical femoral tunnels [7, 20, 24, 41]. Additional studies have also examined how knee flexion angle affects femoral tunnel length, and recommend a high knee flexion angle to maximise tunnel length [36]. While prior studies have sought to determine the ideal FTL, there lacks consensus in the literature with suggested lengths ranging from 15 mm to 30 mm [34]. Further, most of the studies investigating femoral tunnel length are cadaveric in nature, limiting their utility in determining postoperative outcomes. Ultimately, although prior studies have assessed femoral tunnel length as an outcome in the context of other surgical variables, there is a paucity of evidence regarding the impact of FTL on clinical and functional outcomes [16, 42].

Thus, the aim of this systematic review is to determine whether femoral tunnel length affects clinical or functional outcomes following primary ACL reconstruction with single‐bundle quadriceps tendon autograft.

## MATERIALS AND METHODS

This systematic review was conducted in accordance with the guidelines set out by the Cochrane Handbook for Systematic Review of Interventions [17] and is reported according to the Preferred Reporting Items for Systematic Reviews and Meta‐Analysis [33].

### Search strategy

A systematic search of three databases via OVID (MEDLINE, EMBASE, and Cochrane) databases was conducted by one reviewer (TB). The search included studies regarding ACL reconstruction with quadriceps tendon autograft published from 2000 to April 12, 2023. The inclusion criteria were: (1) primary ACL reconstruction with quadriceps tendon autograft, (2) femoral tunnel length reported, (3) single‐bundle quadriceps tendon autograft with or without bone plug, (4) human subjects, (5) skeletally mature patients, (6) functional and clinical outcome data reported, (7) levels of evidence I–IV and (8) studies published in English. The exclusion criteria were: (1) cadaveric or animal studies, (2) review papers or technique papers without outcomes (3) conference abstracts, (4) revision ACL reconstruction, (5) multiligamentous studies, (6) biomechanic papers, (7) double‐bundle quadriceps tendon autograft, (8) grafts other than quadriceps tendon autograft, and (9) no follow‐up/outcomes data reported. The comprehensive search strategy can be found in Appendix A.

### Study screening

The titles and abstracts of all identified studies from the initial search were screened by two authors independently (TB and AV) with reference to the inclusion and exclusion criteria. Disagreements from title and abstract screening continued to the full‐text review stage. Disagreements from the full‐text screening were resolved by consulting a third senior author (HAK). At both the title and abstract stage and full‐text stage, kappa scores were calculated to determine inter‐reviewer agreement.

### Data abstraction

Each reviewer abstracted data from half of the included studies, while the other reviewer assessed for accuracy. Data was abstracted into Google Sheets in preparation for statistical analysis (Google LLC). The following data was abstracted where available: study characteristics (author, publication year, level of evidence, study design and mean follow‐up), patient characteristics (number of patients, sex and mean age), surgical technique, femoral tunnel length, subjective and objective clinical outcomes. The objective outcomes abstracted included (1) Lachman test, (2) anteroposterior (AP) side‐to‐side difference, (3) anterior tibial translation (KT‐1000) and (4) range of motion. The subjective clinical outcomes abstracted included (1) International Knee Documentation Committee (IKDC) score and (2) Lysholm score. Additional outcomes included the pivot‐shift test, re‐rupture rates, and complications.

### Quality assessment

All included studies were non‐randomised, therefore the Methodological Index for Non‐randomised Studies (MINORS) was used to assess the quality of all studies. Two authors (TB and AV) assessed each study independently, and the resulting scores were averaged (Table 1).

**Table 1:** Study characteristics.

Author (year)	Country	Study design	LOE	Patients	Mean age, years	Female, %	Mean follow‐up, months (min follow‐up, months)	Femoral tunnel length, mm	Average MINORS score
S‐FT									
Akoto (2012) [1]	Germany	Case series	IV	30	31*	15*	12 (NR)	20	7.5**
Brinkman (2023) [6]	United States	Retrospective comparison study	III	37	23.4	54	69.9 (NR)	25	19
Chen (2006) [8]	Taiwan	Case series	IV	34	26	35	62 (NR)	20	8**
Karpinski (2021) [22]	Germany	Cohort study	II	25	31.7	64	NR (24)	25	11**
*Weighted mean*					*27.6*	*41.6*	*50*		
*Range*					*23.4–31.7*	*15–64*	*12–69.9*		
L‐FT									
Gorschewsky (2007) [13]	Switzerland	Cohort study	II	193	33	37	29 (NR)	30	7.5**
Kim (2009) – SB vs. DB [21]	Korea	Retrospective comparison study	III	28	26.1	21	24 (NR)	40	18.5
Kim (2009) – QTB vs. PTB [25]	Korea	Retrospective comparison study	III	21	27.1	16	24–36 (range)	30	17.5
*Weighted mean*					*31.7*	*33*	*28.2*		
*Range*					*26.1–33*	*16–37*	*23–35*		

Abbreviations: L‐FT, long femoral tunnel; LOE, level of evidence; MINORS, Methodological Index for Non‐randomised Studies; S‐FT, short femoral tunnel.

* Mean age calculated based on total initial # of patients without accounting for exclusions.

** Noncomparative studies, MINORS scored out of 16 points. The remainder of studies are comparative, scored out of 24 points.

### Statistical analysis

Based on the reported femoral tunnel length, the included studies were divided into two separate groups. The short femoral tunnel length group (S‐FT) included those reporting a femoral tunnel length of exactly 25 mm or less. The long femoral tunnel length group (L‐FT) included the studies reporting a femoral tunnel length greater than 25 mm. Due to a lack of consensus in prior research regarding the ideal femoral tunnel length, a cutoff length of 25 mm was selected for this review to ensure a comparable distribution of patients across both S‐FT and L‐FT groups.

Given that there was significant heterogeneity between studies a meta‐analysis was not conducted. Across studies, there was both methodological and clinical heterogeneity with variations in surgical technique and variations in the clinical outcomes and complications reported. Therefore, weighted means and ranges were calculated for each subjective and objective clinical outcome, for all studies as well as each group (S‐FT and L‐FT) individually. Furthermore, all subjective and objective clinical outcomes were presented in tables. All statistical analysis was conducted in R (RStudio).

## RESULTS

### Literature search

**Figure 1:**
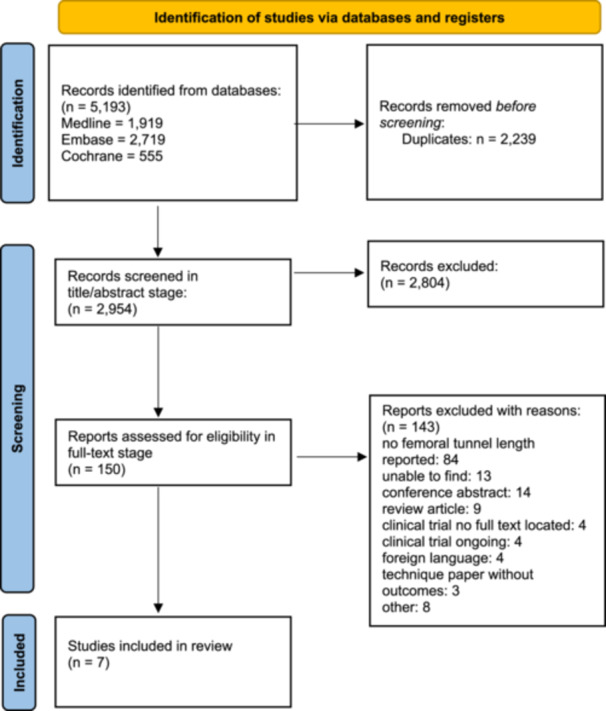
Outline of a systematic search strategy. PRISMA 2020 flow diagram for new systematic reviews which included searches of databases and registers only. *From*: Page MJ, McKenzie JE, Bossuyt PM, Boutron I, Hoffmann TC, Mulrow CD, et al. The PRISMA 2020 statement: an updated guideline for reporting systematic reviews. BMJ 2021;372:n71. doi:10.1136/bmj.n71.

Initially, 5193 studies were identified from a search of MEDLINE, EMBASE, and Cochrane databases with seven studies included in the final analysis (Figure 1) [1, 6, 8, 13, 21, 22, 25]. Agreement was substantial at the title and abstract screening (*κ* = 0.7, 95% confidence interval [CI] 0.7–0.8), and full‐text screening featured almost perfect agreement (*κ* = 0.9, 95% CI 0.8–1.0).

### Study quality

The average MINORS score was found to be 18.3 (±0.8) for comparative studies, denoting moderate quality, and 8.5 (±1.7) for non‐comparative studies, denoting poor quality (Table 1) [40].

### Study characteristics

The included studies were comprised of 368 patients. The mean age of the included patients was 30.3 years (range: 23.4–34 years). Mean postoperative follow‐up time was 35.0 months (range: 12.0–69.9 months). One study did not report follow‐up times (Table 1) [22].

### Surgical technique

In the S‐FT group, the majority of studies utilised the anteromedial portal technique (92 patients) [1, 6, 22], while one study used the transtibial technique (34 patients) [8]. In the L‐FT group, all studies reported using the transtibial technique (242 patients) [13, 21, 25] (Table 2). The range of femoral tunnel lengths reported was 20–40 mm.

**Table 2:** Surgical and fixation techniques.

Authors	Femoral tunnel drilling technique	Femoral tunnel length	QT autograft length, mm	QT autograft diameter, mm ± SD	Patellar bone block length, mm	Femoral fixation technique	Tibial fixation technique
S‐FT							
Akoto (2012)	Anteromedial portal	20	50	NR	20	Press‐fit	Split bone wedge + sutures tied over bone bridge + distal bone plug press‐fit
Brinkman (2023)	Anteromedial portal	25	90	96 ± 6.1	NR	Interference screw (biocomposite)	Interference screw (biocomposite)
Chen (2006)	Transtibial	20	NR	NR	20	Interference screw	sutures tied to bicortical screw and washer
Karpinski (2021)	Anteromedial portal	25	65+	10	15	Press‐fit	Interference screws + tied over button with nonresorbable polyester sutures
L‐FT							
Gorschewsky (2007)	Transtibial	30	90	NR	25	Group 1: Absorbable interference screw	Group 1: Absorbable interference screw
						Group 2: Absorbable cross‐pins	Group 2: Absorbable cross‐pins
Kim (2009) ‐ SB vs. DB	Transtibial	40	55	9	20	Absorbable interference screw	Absorbable interference bioscrew
Kim (2009) ‐ QTB vs. PTB	Transtibial	30	55	9	20	Absorbable interference screw	Absorbable interference bioscrew

Abbreviations: DB, double bundle; L‐FT, long femoral tunnel; NR, not reported; PTB, patellar tendon bone; QT, quadriceps tendon; QTB, quadriceps tendon bone; SB, single bundle; S‐FT, short femoral tunnel.

### Subjective clinical outcomes

Evaluated subjective clinical outcomes were IKDC scores and Lysholm scores (Appendix B). Six studies reported IKDC scores with three reporting both preoperative and postoperative scores. Postoperative IKDC scores in the S‐FT group ranged from 94% to 96.7% [1, 8]. One S‐FT study reported a mean IKDC score of 90.7 ± 6 postoperatively [6]. Postoperative IKDC scores in the L‐FT group ranged from 85% to 93% [13, 21, 25]. Preoperative Lysholm scores in the S‐FT group ranged from 52 to 61.4, and 88.6–93 postoperatively [6, 8, 22]. The range in the L‐FT group was 75.8–77.6 preoperatively and 89–94 postoperatively [13, 21, 25] (Table 3). The statistical significance of these differences was not reported in the studies.

**Table 3:** Preoperative and postoperative subjective clinical outcomes.

IKDC score (Grade A/B, C/D), *n* (%)	Preoperative	Postoperative	*p*‐Value
S‐FT			
Akoto (2012)	NR	A/B: 29 (96.7) C/D: 1 (3.3)	
Chen (2006)	A/B: 0 (0) C/D: 34 (100)	A/B: 32 (94) C/D: 2 (6)	NR
*Weighted mean, %*	*A/B: 0* *C/D: 100*	*A/B: 95.1* *C/D: 4.7*	
*Range*	*A/B: 0‐0* *C/D: 100‐100*	*A/B: 94‐96.7* *C/D: 3.3‐6*	
L‐FT			
Gorschewsky (2007) – pins	NR	A/B: NR (93) C/D: NR (7)	
Gorschewsky (2007) – screws	NR	A/B: NR (85) C/D: NR (15)	
Kim (2009) – SB vs. DB	A/B: 8 (28.6) C/D: 20 (71.5)	A/B: 24 (85.7) C/D: 4 (14.3)	NR
Kim (2009) – QTB vs. PTB	A/B: 5 (23.8) C/D: 16 (76.2)	A/B: 18 (85.7) C/D: 3 (14.3)	NR
*Weighted mean, %*	*A/B: 26.5* *C/D: 73.5*	*A/B: 88.4* *C/D: 11.6*	
*Range*	*A/B: 23.8‐28.6* *C/D: 71.5‐76.2*	*A/B: 85‐93* *C/D: 7‐15*	
Lysholm score (mean ± SD)			
S‐FT			
Brinkman (2023)	52 ± 5.2	92.6 ± 5.3	NR
Chen (2006)	61.4 ± 8.9	93 ± 7.9	NR
Karpinski (2021)	NR	88.6 ± 11.78	
*Weighted mean, %*	*56.5*	*91.7*	
*Range*	*52‐61.4*	*88.6‐93*	
L‐FT			
Gorschewsky (2007) – pins	NR	94 ± NR	
Gorschewsky (2007) – screws	NR	89 ± NR	
Kim (2009) – SB vs. DB	75.8 ± 8.2	91.8 ± 9.7	NR
Kim (2009) – QTB vs. PTB	77.6 ± NR	90.1 ± NR	NR
*Weighted mean, %*	*76.6*	*91.5*	
*Range*	*75.8‐77.6*	*89‐91.8*	

Abbreviations: IKDC, International Knee Documentation Committee; L‐FT, long femoral tunnel; NR, not reported; S‐FT, short femoral tunnel.

### Objective clinical outcomes

Included objective outcomes were the Lachman test, AP side‐to‐side difference, anterior tibial translation, range of motion, pivot shift test, and one leg hop test (Appendix B).

One S‐FT study and one L‐FT study reported preoperative Lachman scores [8, 21] (Table 4). Two S‐FT studies reported a weighted average of 99% of patients had Grade I/II Lachman postoperatively [1, 8]. Between the two L‐FT studies reporting postoperative Lachman scores, 100% of patients had Grade I/II laxity [21, 25]. A single study in the S‐FT group reported preoperative AP side‐to‐side difference [22] (Table 4). Postoperatively, four studies total reported AP side‐to‐side difference with two studies from each group. The S‐FT group had a weighted mean of 1.6 mm while the L‐FT group had a weighted mean of 2.7 mm [1, 21, 22, 25].

**Table 4:** Preoperative and postoperative objective clinical outcomes.

Lachman test (grade I:II:III:IV), *n* (%)	Preoperative	Postoperative	*p*‐Value
S‐FT			
Akoto (2012)	NR	30:0:0:0 (100:0:0:0)	
Chen (2006)	0:0:24:10 (0:0:71:29)	30:3:1:0 (88:9:3:0)	NR
*Weighted mean, %*	*(0:0:71:29)*	*(94:5:1:0)*	
L‐FT			
Kim (2009) – SB vs. DB	2:21:5:0 (7:75:18:0)	27:1:0:0 (96:4:0:0)	NR
Kim (2009) – QTB vs. PTB	NR	19:2:0:0 (90:10:0:0)	
*Weighted mean, %*	*(7:75:18:0)*	*(93:7:0:0)*	
AP side‐to‐side difference, mm (mean ± SD)			
S‐FT			
Akoto (2012)	NR	1.6 ± 1.1	
Karpinski (2021)	6.48 ± 2.00	1.56 ± 1.56	NR
*Weighted mean, %*	*6.48*	*1.58*	
*Range*			
L‐FT			
Kim (2009) – SB vs. DB	NR	2.64 ± 1.62	
Kim (2009) – QTB vs. PTB	NR	2.79 ± 1.32	
*Weighted mean, %*		*2.7*	
*Range*		*2.64–2.79*	
Anterior tibial translation, mm (mean ± SD)			
S‐FT			
Chen (2006)	11.88 ± 1.09	1.74 ± 1.80	<0.01
L‐FT			
Gorschewsky (2007) – pins	NR	0.71 ± NR	
Gorschewsky (2007) – screws	NR	0.69 ± NR	
*Weighted mean, %*		*0.70*	
*Range*		*0.69–0.71*	
Pivot shift test (Grade 0:1:2:3), %			
S‐FT			
Akoto (2012)	NR	83.3:3.3:13.3:0	
Karpinski (2021)	NR	100:0:0:0	
L‐FT			
Kim (2009) – SB vs. DB	0:3.6:85.7:10.7	85.7:10.7:3.6:0	NR

Abbreviations: L‐FT, long femoral tunnel; NR, not reported; S‐FT, short femoral tunnel.

Anterior tibial translation was reported preoperatively and postoperatively in one S‐FT study, and demonstrated a significant postoperative improvement (*p* < 0.01) [8]. Two studies reported postoperative anterior tibial translation in the L‐FT group, with a range from 0.69 mm to 0.71 mm [13]. Range of motion was exclusively reported postoperatively. In the S‐FT group, full ROM was reported in 87.5%–92.3% of patients [1, 8, 22]. In the L‐FT group, full postoperative ROM was reported in 85.7%–92.8% of patients [13, 21, 25] (Table 5).

**Table 5:** Postoperative range of motion.

Range of motion (ROM)	Full ROM, %	Lack of extension, %	Lack of flexion, %
S‐FT			
Akoto (2012)	92.3	3	3
Brinkman (2023)	NR	NR	3
Chen (2006)	91	6	3
Karpinski (2021)	87.5	8	NR
*Weighted mean, %*	*90.5*	*5.6*	*3*
*Range*	*87.5–92.3*	*3–8*	3–3
L‐FT			
Gorschewsky (2007) – pins	87	11	11
Gorschewsky (2007) – screws	87	11	11
Kim (2009) – SB vs. DB	92.8	4	4
Kim (2009) – QTB vs. PTB	85.7	10	5
*Weighted mean, %*	*87.6*	*10.1*	*9.7*
*Range*	*85.7–92.8*	*4–11*	*4–11*

Abbreviations: L‐FT, long femoral tunnel; NR, not reported; S‐FT, short femoral tunnel.

In total, four studies reported results for the pivot shift test [1, 21, 22, 25]. The two S‐FT studies reported postoperative pivot shift with 83.3% and 100% of patients with Grade 0 pivot shift respectively [1, 22]. One study in the L‐FT group reported both preoperative and postoperative pivot shift results, while the other reported only postoperative [21, 25]. One LFT study reported that two patients had a Grade 2 pivot shift postoperatively, however, the authors did not include this in their total percentage so this was not included in the results in Table 4 [25].

Four studies reported postoperative one‐leg hop test results. One S‐FT study reported a mean of 91.9 ± 8.0 postoperatively [1]. One study in the L‐FT group reported scores separately based on the type of graft fixation [13]. In the pin‐fixation group, 94% of patients were graded normal or nearly normal (75%–100% compared to unoperated leg). In the screw‐fixation group, 94% of patients were graded normal or nearly normal. The other L‐FT study found that 81% of patients demonstrated normal limb symmetry scores postoperatively [21].

### Complications/re‐rupture

In the S‐FT group, the complication rate ranged from 0% to 29% with a weighted mean of 11.94% [1, 6, 8, 22], and from 1% to 14% with a weighted mean of 4.5% in the L‐FT group [13, 21, 25] (Table 6).

**Table 6:** Complications.

Study	Arthrofibrosis	Contralateral Tear	Infection	Donor site morbidity	Crepitus	Stitch abscess	Numbness at tibial incision	Hardware irritation and removal	Total complications, *n* (%)	Re‐rupture, %
S‐FT										
Akoto (2012)	NR	NR	NR	NR	NR	NR	NR	NR	0 (0)	0
Brinkman (2023)	3	2	0	NR	NR	NR	NR	NR	5 (14)	3
Chen (2006)	NR	NR	NR	3	2	2	2	1	10 (29)	0
Karpinski (2021)	NR	NR	0	NR	NR	NR	NR	NR	0 (0)	0
*Weighted mean, %*									*11.9*	*0.8*
*Range*									*0–29*	*0–3*
L‐FT										
Gorschewsky (2007) – pins	NR	NR	NR	2	4	NR	NR	NR	6 (6)	2
Gorschewsky (2007) – screws	NR	NR	NR	0	2	NR	NR	NR	2 (2)	0
Kim (2009) – SB vs. DB	NR	NR	NR	NR	NR	NR	NR	NR	0 (0)	0
Kim (2009) – QTB vs. PTB	NR	NR	NR	2	1	NR	NR	NR	3 (5)	0
*Weighted mean*									*4.5*	*0.9*
*Range*									*0–6*	*0–2*

Abbreviations: L‐FT, long femoral tunnel; NR, not reported; S‐FT, short femoral tunnel.

Only two studies reported re‐ruptures requiring revision ACLR. The rate of re‐rupture ranged from 0% to 2% in the S‐FT group [6], and from 0% to 3% in the L‐FT group [13].

## DISCUSSION

The main finding of this systematic review is that postoperative outcomes following ACLR reconstruction with QT autograft are comparable across both short and long femoral tunnel lengths using a 25 mm tunnel length threshold between groups. Nonetheless, there were trends of slightly superior outcomes in favour of short femoral tunnel length in terms of IKDC score, AP side‐to‐side difference, and range of motion, though a limited sample size of non‐comparative studies prevented a formal meta‐analysis. This review further confirmed that anatomic femoral tunnel drilling is generally associated with shorter femoral tunnel length, while non‐anatomic (i.e., trans‐tibial drilling) techniques resulted in a longer femoral tunnel length. Ultimately, this study demonstrated that very limited clinical evidence exists on femoral tunnel length in the context of ACLR with QT tendon.

This review serves as an important starting point in assessing the impact of femoral tunnel length on subsequent postoperative clinical outcomes. The majority of literature to date surrounding femoral tunnel length has focused mostly on surgical technique, tunnel placement, and minimum required femoral tunnel length in ACLR rather than the impact on clinical outcomes and there exists a lack of consensus in terms of the ideal femoral tunnel length [14, 30, 42]. One study aimed to assess the impact of femoral tunnel length on clinical outcomes and re‐rupture, however due to multiple limitations of the study including a sample size of 71 patients and heterogeneity of surgical techniques, statistical analysis was limited [14]. They did not find any significant differences between the two groups in terms of re‐rupture rates, Lachman test, pivot shift, IKDC, Lysholm, and other clinical outcomes. Similarly to this review, they reported that the transtibial technique resulted in longer femoral tunnel lengths compared to anteromedial portal technique (range: 40–65 mm compared to range: 29–50 mm respectively) [14]. Of note, this study used hamstring tendon grafts and reported the amount of graft within the tunnel rather than the length of the femoral tunnel specifically, limiting its direct applicability for the purposes of this review [14]. Thus, comparative studies with larger sample sizes and differing graft types are needed to further assess the impact of femoral tunnel length on clinical outcomes.

Further, it cannot be overlooked that differing surgical techniques were reported in the included studies, which are known to impact both femoral tunnel length and clinical outcomes [4, 16, 27, 29]. Numerous studies have reported that the anteromedial portal technique results in a more anatomically oriented and shorter graft in comparison to the transtibial technique, which results in a more vertically oriented and longer graft and is associated with suboptimal rotational and translational stability [7, 26, 28, 29]. This finding is consistent with the studies included in this review, as all studies in the L‐FT group reported using the transtibial technique and three of four studies in the S‐FT group reported using the anteromedial portal technique [1, 6, 8, 13, 21, 22, 25].

Trends in subjective and objective outcomes demonstrated by this review may be explained by the different tunnel techniques utilised, with slightly superior outcomes in the S‐FT group, of which most studies used the anteromedial portal technique. While both groups demonstrated high rates of full postoperative range of motion, the S‐FT group was slightly higher. Furthermore, the S‐FT group demonstrated slightly superior postoperative AP side‐to‐side differences. In terms of subjective clinical outcomes, IKDC scores overall were slightly in favour of the S‐FT group. Similar findings were reported in a review that found superior outcomes in patients undergoing ACLR with the anteromedial portal technique compared to the transtibial technique [9]. In this review, the S‐FT group did experience a significantly higher rate of all‐cause complications with a weighted mean of 12% compared to the weighted mean of the L‐FT group of 4.5%. However, due to study heterogeneity, statistical significance of this difference could not be assessed. Notably, the majority of complications in the S‐FT group were described in a single study and interestingly, this was also the only study that used the transtibial surgical technique, possibly suggesting that superior outcomes are associated with the anteromedial portal technique [8]. Although it is possible that superior outcomes are related to short femoral tunnel length, this conclusion cannot be drawn in this review as surgical technique is a confounding factor to be controlled for. Thus, femoral tunnel length should be assessed independently of surgical technique in future studies.

The rates of re‐rupture in this review were found to be comparably low in both groups. In the S‐FT group there was one total case of re‐rupture across all studies [1, 6, 8, 22]. In comparison, there were two total cases of re‐rupture reported in one L‐FT study, which were attributed to injury during sport [13, 21, 25]. These low rates of re‐rupture are consistent with existing literature regarding ACLR with QT autograft, with one study describing a rate of re‐rupture of 2.5% in ACLR with QT autograft compared to 8.7% in patients receiving hamstring tendon autograft [19]. Similarly, another study reported a re‐rupture rate of 10.9% in the hamstring tendon group, reinforcing that QT is a viable option in ACLR, particularly in terms of rates of re‐rupture, which could explain the low rates seen in this review [6]. Additionally, numerous studies have demonstrated no difference in rates of re‐rupture between quadriceps tendon autografts and bone patellar tendon‐bone autografts [3, 18, 35]. Therefore, in consideration of further direction for this review one could assess the impact of femoral tunnel length in patients receiving autografts other than QT.

There are limitations of this systematic review that should be noted. First, the level of evidence of all studies ranged from level II to level IV, preventing meta‐analysis of the results. Therefore, conclusions drawn regarding the data presented should be done with consideration of the quality of evidence. There are further limitations in terms of generalisability, as the impact of femoral tunnel length on clinical outcomes was evaluated only for ACL reconstruction with single bundle quadriceps tendon autograft. Furthermore, multiple surgical and graft fixation techniques were described, both of which have the potential to impact clinical outcomes. There was also heterogeneity in the reporting of multiple clinical outcomes preventing statistical analysis of these results. Ultimately, this review highlights the need for focused research on the impact of femoral tunnel length on outcomes following ACLR. Several study designs may be leveraged to this phenomenon ranging from in vitro cadaveric studies to surveys across thought leaders in the field, as is often conducted when clinical equipoise exists. Lastly, we recommend the standardised reporting of femoral tunnel length in future ACLR literature to allow for larger, clinically impactful review studies [12, 43, 44].

## CONCLUSION

Both short and long femoral tunnel lengths demonstrated comparable postoperative clinical and functional outcomes following primary ACLR with single bundle quadriceps tendon autograft. There were slightly superior outcomes reported with short femoral tunnel length, however this may have been confounded by the variation in surgical technique used.

## AUTHOR CONTRIBUTIONS

**Tess Bracken:** Screening; data extraction; manuscript drafting and editing. **Alexandre Veilleux:** Screening; data extraction; manuscript drafting. **Hassaan Abdel Khalik:** Idea conception; statistical analysis; supervision; manuscript editing. **Jansen Johnson:** Supervision; manuscript editing. **Darren de SA:** Supervision; manuscript editing.

## CONFLICT OF INTEREST STATEMENT

The authors declare no conflict of interest.

## ETHICS STATEMENT

The authors have no relevant ethical disclosures to report.

## Data Availability

Data may be provided upon reasonable request to the corresponding author. The data utilised for this article is available through open access journals or McMaster University's Health Science Library access when articles required subscription.

## Appendix A Search strategy

1. exp anterior cruciate ligament/
2. anterior cruciate ligament.mp
3. acl.mp
4. 1 or 2 or 3
5. reconstruction.mp or anterior cruciate ligament reconstruction/
6. recon*.mp
7. repair*.mp
8. 5 or 6 or 7
9. quadriceps tendon.mp or quadriceps tendon/
10. quadriceps autograft.mp
11. quad*.mp
12. 9 or 10 or 11
13. 4 and 8 and 12
14. limit 13 to yr = *2000‐current*

## Appendix B Subjective and objective clinical outcomes

Subjective Clinical Outcomes

The International Knee Documentation Committee (IKDC) subjective knee evaluation form was first published in 1993 as a standardised questionnaire for evaluating improvement or deterioration of function, symptoms, and sporting activities due to knee injuries. It consists of three domains and the possible scoring range is 0–100, where 100 represents the absence of symptoms and no functional limitations or limit to sporting activities [11].

The Lysholm Knee Scoring Scale was first published in 1982 and subsequently revised in 1985. It is used to evaluate outcomes of knee ligament surgery in terms of knee instability. It is scored from 0–100 where 100 represents no symptoms or disability. 95–100 is excellent, 84–94 is good, 65–83 is fair and <65 is poor [11].

Objective Clinical Outcomes

There was heterogeneity between studies in the grading of the Lachman test with some studies including Grade 0 as <3 mm translation. For the purposes of this review, all Lachman test results were graded on a scale of Grade I, II, III, and IV, with I representing 0–5 mm of translation, II representing 6–10 mm, III representing 11–15 mm, and IV representing >15 mm of translation.

There was also heterogeneity in the reporting of the Pivot Shift Test. For the purposes of this review, negative pivot shift test was treated as equivalent to Grade 0. Descriptions of the one leg hop test grading differed by studies and was therefore reported qualitatively in the results. Studies that reported anterior tibial translation utilised the KT‐1000 arthrometer (MEDmetric Corporation) performed with a maximum manual or standard force of 134 N.
